# ﻿Further notes on the genus *Eurhaphidophora* Gorochov, 1999 (Orthoptera, Rhaphidophoridae) with description of a new species from China

**DOI:** 10.3897/zookeys.1211.128308

**Published:** 2024-09-02

**Authors:** Qidi Zhu, Fuming Shi

**Affiliations:** 1 College of Agronomy, Jiangxi Agricultural University, Nanchang 330045, China Jiangxi Agricultural University Nanchang China; 2 Key Laboratory of Zoological Systematics and Application of Hebei Province, College of Life Sciences, Hebei University, Baoding 071002, China Hebei University Baoding China

**Keywords:** Cave crickets, morphology, new species, new synonymy, taxonomy

## Abstract

This paper revises the genus *Eurhaphidophora* from China and describes a new species, i.e., *Eurhaphidophoradulongjiangensis* Zhu & Shi, **sp. nov.** The females of *Eurhaphidophoratarasovidoitungensis* Dawwrueng, Gorochov & Suwannapoom, 2020 and *Eurhaphidophorafossa* Lu, Huang & Bian, 2022 are described for the first time. Moreover, *Eurhaphidophoracurvata* Lu, Huang & Bian, 2022, **syn. nov.** is considered as a new synonym of *Eurhaphidophorapawangkhananti* Dawwrueng, Gorochov & Suwannapoom, 2020. Images illustrating the morphology of these species are provided.

## ﻿Introduction

Gorochov (1999) established the genus *Eurhaphidophora* Gorochov, 1999 and assigned *Eurhaphidophoranataliae* Gorochov, 1999 from Vietnam as type species. Thereafter, nine species were described from China, Vietnam, Laos, Thailand and Malaysia (Gorochov 2010, 2011, 2012). Later, *E.truncata* Bian & Shi, 2016, *E.curvata* Lu, Huang & Bian, 2022 and *E.fossa* Lu, Huang & Bian, 2022 were published from China (Bian and Shi 2016; Lu et al. 2022), while *E.pawangkhananti* Dawwrueng, Gorochov & Suwannapoom, 2020, *E.tarasovidoitungensis* Dawwrueng, Gorochov & Suwannapoom, 2020 and *E.apicoexcisa* Dawwrueng, Gorochov, Pinkaew & Vitheepradit, 2023 were discovered from Thailand (Dawwrueng et al. 2020, 2023).

Up to now, the genus *Eurhaphidophora* includes fifteen species, four of which are recorded from China. Here, we describe a new species *E.dulongjiangensis* Zhu & Shi, sp. nov. from China, describe the females of *E.tarasovidoitungensis* Dawwrueng, Gorochov & Suwannapoom, 2020 and *E.fossa* Lu, Huang & Bian, 2022 for the first time, and propose *E.curvata* Lu, Huang & Bian, 2022, syn. nov. to become a new synonym of *Eurhaphidophorapawangkhananti* Dawwrueng, Gorochov & Suwannapoom, 2020.

## ﻿Materials and methods

Specimens were collected by hand at night and preserved in 75% ethanol. The genitalia were dissected with an insect needle and then put in 10% KOH solution to clean the tissue. Images were taken with a Zeiss AxioCam ICc5 digital camera attached to a Zeiss Stereo Discovery V12 microscope and edited with ADOBE PHOTOSHOP 2022. With regard to the scheme of arrangement of spines on the tibiae and hind basitarsus we follow Gorochov and Storozhenko (2015) and for measurements we follow Zhu et al. (2022). The type specimen is deposited in the Museum of Hebei University, Baoding, China (**HBU**).

## ﻿Results

### ﻿Genus Eurhaphidophora

Taxon classificationAnimaliaOrthopteraRhaphidophoridae

Gorochov, 1999

14A15F23-622D-5260-9E2B-27E80F443FD5

#### Type species.

*Eurhaphidophoranataliae* Gorochov, 1999, by original designation.

#### Diagnosis.

Body medium-sized in Rhaphidophorinae. Seventh and eighth abdominal tergites of male with a small posterior median projection that is nearly rounded or angular. Posterior margin of ninth abdominal tergite of male provided with a large median process. Male epiproct simple. Male genitalia membranous. Lateral lobes of dorso-median blade large, almost entirely covering central lobe of this blade.

**Figure 1.**
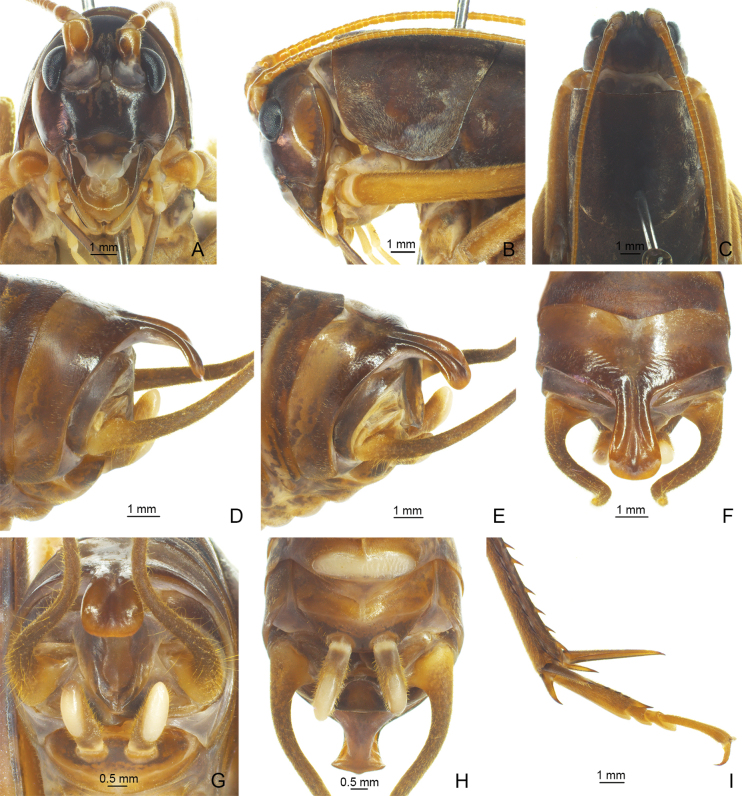
*Eurhaphidophoradulongjiangensis* Zhu & Shi, sp. nov. **A–I** ♂ **A–C** head and pronotum **A** frontal view **B** lateral view **C** dorsal view **D–H** apex of abdomen **D** lateral view **E** apico-lateral view **F** dorsal view **G** apical view **H** ventral view **I** hind tarsus in lateral view.

#### Distribution.

China, Laos, Malaysia, Thailand and Vietnam.

### ﻿ Eurhaphidophora dulongjiangensis

Taxon classificationAnimaliaOrthopteraRhaphidophoridae

Zhu & Shi sp. nov.

C8523007-929C-5C26-8C59-01197BF62653

https://zoobank.org/FAE6DAB0-1028-40DD-A5A2-1F8BA6D2411A

Figs 1 , 2A, B

#### Type material.

***Holotype.*** ♂, China: Yunnan Province, Gongshan County, Dulongjiang Town, Bapo Village, 27.7418°N, 98.3561°E, alt. 1610 m, 9.VII.2021, Shengchuan Yang leg.

#### Diagnosis.

The new species can be distinguished from other congeneric species by the shape of the male epiproct and the ninth abdominal tergite. The ninth abdominal tergite of the male has a long posteromedian process, basal half narrow with a longitudinal median furrow, lateral sides raised into ridges; apical half slightly broadened and curved downwards, with a carina in midline, apex truncate. Male epiproct linguiform, concave on ventral side, apical area slightly protruding.

**Figure 2.**
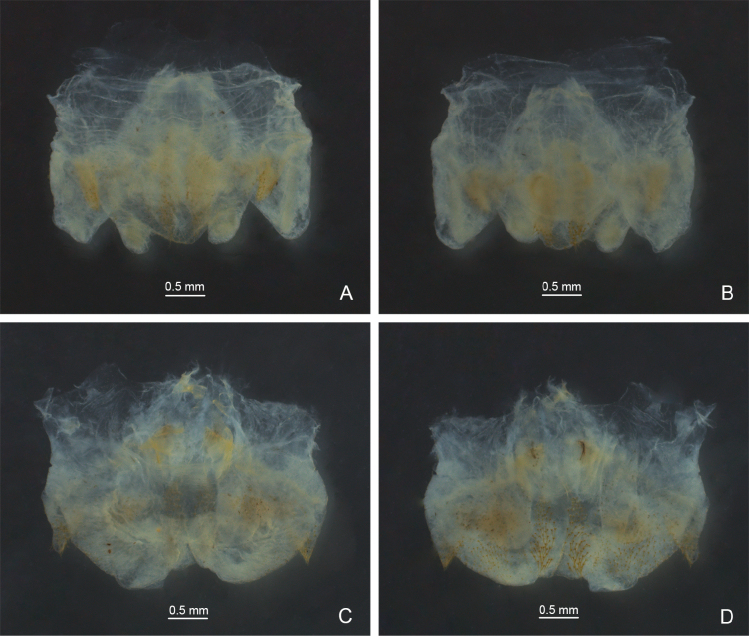
Male genitalia of *Eurhaphidophora* spp. **A, C** dorsal view **B, D** ventral view **A, B***Eurhaphidophoradulongjiangensis* Zhu & Shi, sp. nov. **C, D***Eurhaphidophoratarasovidoitungensis* Dawwrueng, Gorochov & Suwannapoom, 2020.

#### Description.

**Male.** Body medium-sized. Fastigium verticis with rostral tubercles, pressed to each other and divided by a narrow and deep furrow, pointing forwards. Eyes ovoid, protruding forwards; lateral ocelli large and circular, occupying basal 2/3 of lateral surface of rostral tubercles; median ocellus slightly smaller, oval, located between antennal sockets. Pronotum long, anterior margin straight, posterior margin arcuate; lateral lobe longer than high, ventral margin arc-shaped. Mesonotum and metanotum short, posterior margin of mesonotum arcuate, posterior margin of metanotum straight. Fore coxa with one small spine. Internal genicular lobe of fore femur with one long spine; internal and external genicular lobes of mid femur each with one long spine; hind femur with one inner spine on ventral surface, internal genicular lobe with one small spine. Tibia and hind basitarsus with following armament – ve, vi, ve, v2a / de, d~2, d2a, ve, ve, v2a / d20e–18i (d22e–20i), d2sa, 6a / d3c, dac. Posterior margin of eighth abdominal tergite angularly projecting. Ninth abdominal tergite with long posteromedian process, basal half narrow with a longitudinal median furrow, lateral sides raised into ridges; apical half slightly broadened and curved downwards, with a carina in midline, apex truncate. Epiproct linguiform, concave ventrad, apical area slightly protruding; paraproct nearly triangular in lateral view. Cercus narrow, conical, apex acute. Subgenital plate transverse and broad, posterior margin straight. Stylus cylindrical, apex rounded, inserted on posterolateral area of subgenital plate. Genitalia membranous. **Female.** Unknown.

***Coloration*.** Body light brown. Face, fastigium verticis and eyes black; ocelli pale. Thoracic tergites brown.

***Measurements (mm).*** Body length: ♂29.60; length of pronotum: ♂7.44; length of fore femur: ♂9.36; length of hind femur: ♂20.18; length of hind tibia: ♂18.34; length of hind basitarsus: ♂3.50.

#### Etymology.

The name of the new species derives from the type locality.

#### Distribution.

China (Yunnan).

### ﻿ Eurhaphidophora tarasovi doitungensis

Taxon classificationAnimaliaOrthopteraRhaphidophoridae

Dawwrueng, Gorochov & Suwannapoom, 2020

FC1C9BDF-ABED-534A-9F05-966DB2B603C8

Figs 2C, D , 3

Eurhaphidophora tarasovi doitungensis Dawwrueng, Gorochov & Suwannapoom, 2020. In: Dawwrueng, Gorochov, Tanomtong and Suwannapoom 2020: 240.

#### Material examined.

1♂1♀, China: Yunnan Province, Lvchun County, Banpo Town, 22.6517°N, 102.1236°E, alt. 1073 m, 17.VIII.2023, Mengjia Zheng leg.

**Figure 3.**
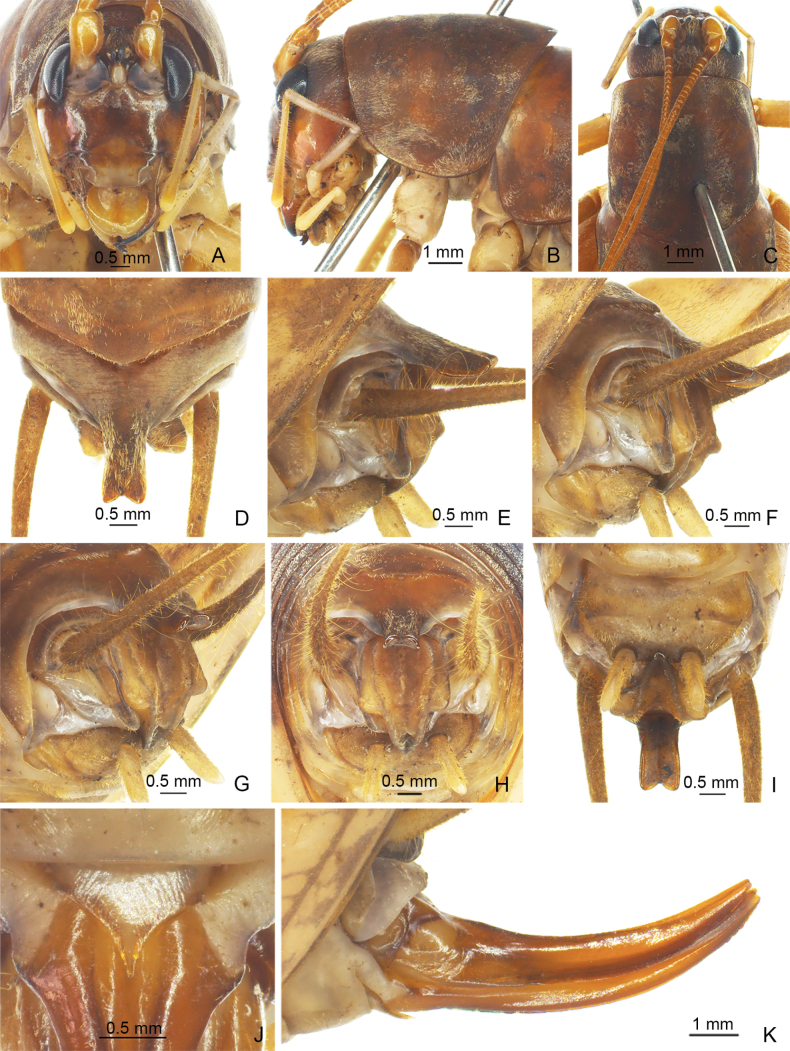
*Eurhaphidophoratarasovidoitungensis* Dawwrueng, Gorochov & Suwannapoom, 2020 **A–I** ♂ **A–C** head and pronotum **A** frontal view **B** lateral view **C** dorsal view **D–I** apex of abdomen **D** dorsal view **E** lateral view **F, G** apico-lateral view **H** apical view **I** ventral view **J, K** ♀ **J** subgenital plate **K** ovipositor in lateral view.

#### Description.

**Male.** Body medium-sized. Fastigium verticis with rostral tubercles, pressed to each other and divided by a narrow and deep furrow, pointing forwards. Eyes ovoid, protruding forwards; lateral ocelli large and circular, occupying basal 2/3 of lateral surface of rostral tubercles; median ocellus slightly smaller, oval, located between antennal sockets. Pronotum long, anterior margin straight, posterior margin arcuate; lateral lobe longer than high, ventral margin arc-shaped. Mesonotum and metanotum short, posterior margin of mesonotum arcuate, posterior margin of metanotum straight. Fore coxa with one small spine. Internal genicular lobe of fore femur with one long spine; internal and external genicular lobes of mid femur each with one long spine; internal genicular lobe of hind femur with one small spine. Tibia and hind basitarsus with following armament – ve, vi, ve, v2a / d~2, d2a, ve, ve, v2a / d17e–17i (d21e–19i), d2sa, 6a / d2c (d3c), dac. Posterior margin of eighth abdominal tergite angularly projecting. Ninth abdominal tergite with long posteromedian process, parallel on both sides, lateral margin bent downwards, apical area with a wide notch. Epiproct with longitudinal median concavity on dorsal surface, basal half with a pair of angular lateral lobes, apical half linguiform, curved downwards and forwards; paraproct nearly triangular in lateral view. Cercus slender, conical, apex acute. Subgenital plate transverse and broad, posterior margin straight. Stylus cylindrical, apex rounded, inserted on posterolateral area of subgenital plate. Genitalia membranous. **Female.** Posterior margin of ninth abdominal tergite slightly convex. Epiproct lingulate. Ovipositor short, slightly curved upwards, apical area of ventral margin denticulate. Subgenital plate triangular, apex acute.

***Coloration*.** Body light brown. Face, fastigium verticis and thoracic tergites brown. Eyes black, ocelli pale.

***Measurements (mm)*.** Body length: ♂23.70, ♀18.10; length of pronotum: ♂6.48, ♀6.48; length of fore femur: ♂7.60, ♀7.44; length of hind femur: ♂18.26, ♀17.66; length of hind tibia: ♂16.86, ♀15.90; length of hind basitarsus: ♂2.66, ♀2.96; length of ovipositor: 8.26.

#### Distribution.

China (Yunnan); Thailand.

#### Remarks.

The species is newly recorded from China and the female is described for the first time.

### ﻿ Eurhaphidophora pawangkhananti

Taxon classificationAnimaliaOrthopteraRhaphidophoridae

Dawwrueng, Gorochov & Suwannapoom, 2020

C7FBCD75-228B-579C-9EC5-310DAABD691D

Figs 4 , 5 , 6A, B

Eurhaphidophora pawangkhananti Dawwrueng, Gorochov & Suwannapoom, 2020. In: Dawwrueng, Gorochov, Tanomtong and Suwannapoom 2020: 242.
Eurhaphidophora curvata Lu, Huang & Bian, 2022, syn. nov.

#### Material examined.

China: • Yunnan Province, 1♂1♀, Puer City, Meizihu Park, 22.7594°N, 100.9963°E, alt. 1302 m, 20.VIII.2019, Qidi Zhu leg.; • 4♂♂2♀♀, Puer City, Yixiang Town, 22.7487°N, 101.0563°E, alt. 1470 m, 22.VIII.2019, Qidi Zhu leg.; • 12♂♂20♀♀, Puer City, Meizihu Park, 22.7594°N, 100.9963°E, alt. 1302 m, 19.VIII.2023, Jie Su leg.

**Figure 4.**
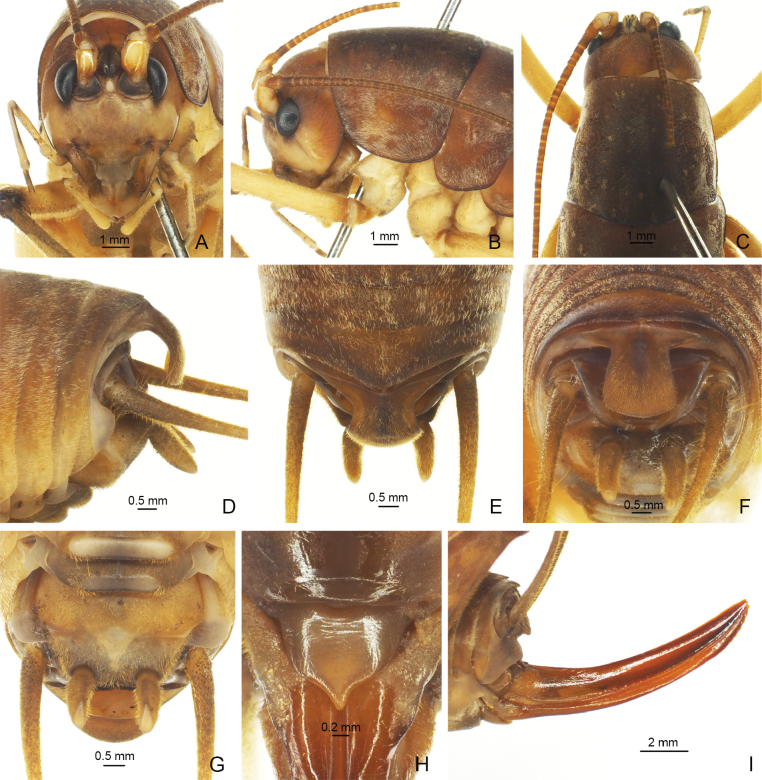
*Eurhaphidophorapawangkhananti* Dawwrueng, Gorochov & Suwannapoom, 2020 **A–G** ♂ **A–C** head and pronotum **A** frontal view **B** lateral view **C** dorsal view **D–G** apex of abdomen **D** lateral view **E** dorsal view **F** apical view **G** ventral view **H, I** ♀ **H** subgenital plate **I** ovipositor in lateral view.

**Figure 5.**
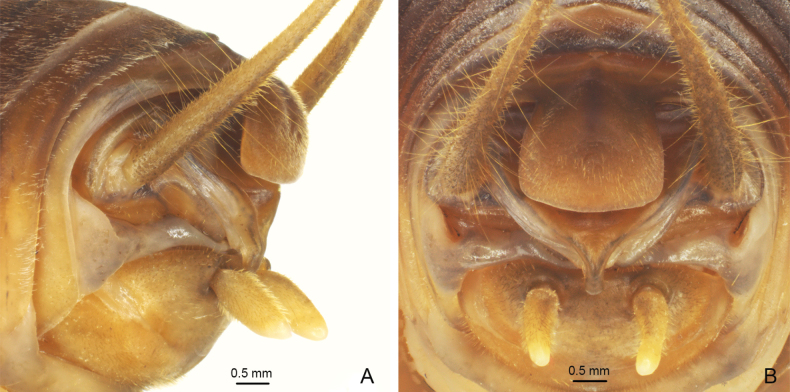
*Eurhaphidophorapawangkhananti* Dawwrueng, Gorochov & Suwannapoom, 2020 **A, B** apex of male abdomen **A** apico-lateral view **B** apical view.

**Figure 6.**
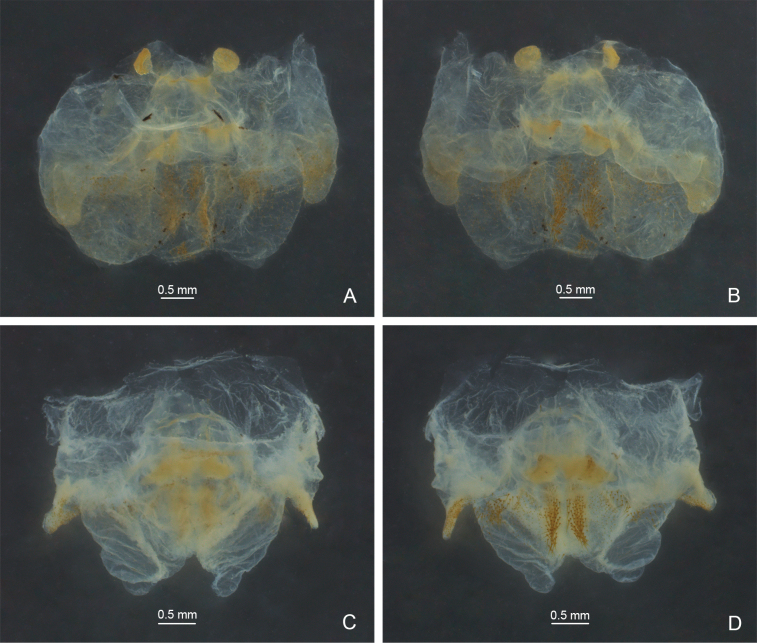
Male genitalia of *Eurhaphidophora* spp. **A**, **C** dorsal view **B**, **D** ventral view **A, B***Eurhaphidophorapawangkhananti* Dawwrueng, Gorochov & Suwannapoom, 2020 **C, D***Eurhaphidophorafossa* Lu, Huang & Bian, 2022.

#### Description.

**Male.** Body medium-sized. Fastigium verticis with rostral tubercles, pressed to each other and divided by a narrow and deep furrow, pointing forwards. Eyes ovoid, protruding forwards; lateral ocelli large and circular, occupying basal 2/3 of lateral surface of rostral tubercles; median ocellus slightly smaller, oval, located between antennal sockets. Pronotum long, anterior margin straight, posterior margin arcuate; lateral lobe longer than high, ventral margin arc-shaped. Mesonotum and metanotum short, posterior margin of mesonotum arcuate, posterior margin of metanotum straight. Fore coxa with one small spine. Internal genicular lobe of fore femur with one long spine; internal and external genicular lobes of mid femur each with one long spine; internal genicular lobe of hind femur with one small spine. Tibia and hind basitarsus with following armament – ve, (vi), ve, v2a / d~2, d2a, ve, ve, v2a / d18e–18i (d20e–19i), d2sa, 6a / d1c (d4c), dac. Posterior margin of eighth abdominal tergite angularly projecting. Ninth abdominal tergite long and wide, strongly curved downwards, basal half with a short dorso-median ridge, apex nearly truncate. Epiproct cup-shaped, basal half wide, nearly semicircular, apical process narrow, curved downwards and forwards. Cercus slender, conical, apex acute. Subgenital plate transverse and broad, posterior margin straight. Stylus cylindrical, apex rounded, inserted on posterolateral area of subgenital plate. Genitalia membranous. **Female.** Posterior margin of ninth abdominal tergite with small projection. Epiproct lingulate. Ovipositor slightly curved upwards, apical area of ventral margin denticulate. Subgenital plate nearly triangular, apex acute.

***Coloration*.** Body light brown. Eyes black, ocelli pale.

***Measurements (mm)*.** Body length: ♂25.50–26.8, ♀24.68–25.40; length of pronotum: ♂6.54–6.90, ♀6.58–6.60; length of fore femur: ♂7.52–7.80, ♀7.50–7.76; length of hind femur: ♂17.06–17.66, ♀17.38–17.96; length of hind tibia: ♂15.58–15.90, ♀15.02–15.50; length of hind basitarsus: ♂3.22–3.26, ♀2.96–3.20; length of ovipositor: 12.02–12.80.

#### Distribution.

China (Yunnan); Thailand.

#### Remarks.

Dawwrueng et al. (2020) described *E.pawangkhananti* from Thailand. Then, Lu et al. (2022) published *E.curvata* from China and thought it was close to *E.ampla* Gorochov, 2010 and *E.orlovi* Gorochov, 2010. Dawwrueng et al. (2023) compared *E.curvata* to *E.pawangkhananti*, which is very similar to *E.curvata*. The two species can be distinguished by the characteristics of the male epiproct and the subgenital plate. However, the male epiproct of *E.curvata* is also greatly similar to that of *E.pawangkhananti*, which is cup-shaped, broad and rather short with an apical process that is very narrow and slightly curved forward in lateral view (Fig. 5). When the apical part is not fully exposed, the posterior margin of the epiproct appears to be widely rounded (Fig. 4F). Moreover, it is not obvious whether the posterior margin of the male subgenital plate between its styli is convex or almost straight, so it cannot be used as the main distinguishing character. Therefore, we consider *E.curvata* Lu, Huang & Bian, 2022, syn. nov. to be a new synonym of *E.pawangkhananti* Dawwrueng, Gorochov & Suwannapoom, 2020.

### ﻿ Eurhaphidophora fossa

Taxon classificationAnimaliaOrthopteraRhaphidophoridae

Lu, Huang & Bian, 2022

EAC6ED32-366F-55E0-82D9-1B37438071D7

Figs 6C, D , 7

Eurhaphidophora fossa Lu, Huang & Bian, 2022: 394.

#### Material examined.

China: • Yunnan Province, 5♂♂4♀♀, Jinghong City, Gasa Town, 21.9589°N, 100.7678°E, alt. 1340 m, 11.VIII.2019, Qidi Zhu leg.; • 1♂3♀♀, Menghai County, Guomenshan, 22.0610°N, 100.5682°E, alt. 1770 m, 11.VIII.2023, Jie Su and Sheng Gao leg.; • 4♂♂6♀♀, Lvchun County, Banpo Town, 22.6517°N, 102.1236°E, alt. 1073 m, 17.VIII.2023, Mengjia Zheng, Xiaolong Tong and Tianshuo Han leg.

**Figure 7.**
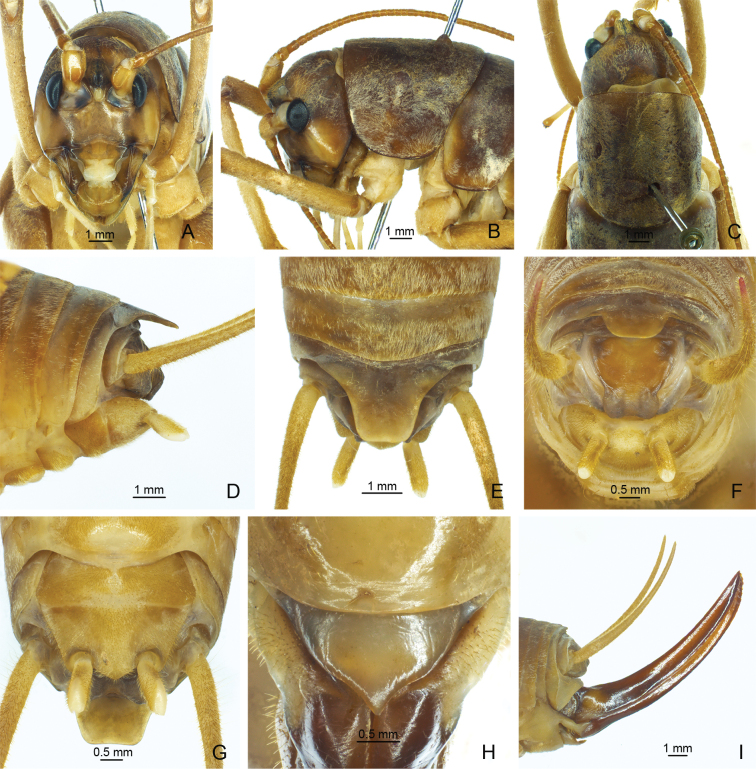
*Eurhaphidophorafossa* Lu, Huang & Bian, 2022 **A–G** ♂ **A–C** head and pronotum **A** frontal view **B** lateral view **C** dorsal view **D–G** apex of abdomen **D** lateral view **E** dorsal view **F** apical view **G** ventral view. **H, I** ♀ **H** subgenital plate **I** ovipositor in lateral view.

#### Description.

**Male.** Body medium-sized. Fastigium verticis with rostral tubercles, pressed to each other and divided by a narrow and deep furrow, pointing forwards. Eyes ovoid, protruding forwards; lateral ocelli large and circular, occupying basal 2/3 of lateral surface of rostral tubercles; median ocellus slightly smaller, oval, located between antennal sockets. Pronotum long, anterior margin straight, posterior margin arcuate; lateral lobe longer than high, ventral margin arc-shaped. Mesonotum and metanotum short, posterior margin of mesonotum arcuate, posterior margin of metanotum straight. Fore coxa with one small spine. Internal genicular lobe of fore femur with one long spine; internal and external genicular lobes of mid femur each with one long spine; internal genicular lobe of hind femur with one small spine. Tibia and hind basitarsus with following armament – ve, vi, ve, v2a / d~2, d2a, ve, ve, v2a / d17e–16i (d19e–19i), d2sa, 6a / d1c (d5c), dac. Posterior margin of eighth abdominal tergite rounded. Ninth abdominal tergite long, trapezoid. Basal 2/3 of epiproct trapezoid, apical 1/3 rectangular, curved downwards; paraproct nearly triangular in lateral view. Cercus narrow, conical, apex acute. Subgenital plate transverse and broad. Stylus cylindrical, apex rounded, inserted in posterolateral area of subgenital plate. Genitalia membranous. **Female.** Posterior margin of ninth abdominal tergite with small projection. Epiproct lingulate. Ovipositor slightly curved upwards, apical area of ventral margin denticulate. Subgenital plate nearly triangular, apex acute.

***Coloration*.** Body light brown. Eyes black, ocelli pale.

***Measurements (mm)*.** Body length: ♂27.76–27.94, ♀28.00–28.60; length of pronotum: ♂6.68–7.20, ♀7.40–7.68; length of fore femur: ♂7.72–7.80, ♀7.80–8.38; length of hind femur: ♂18.26–19.38, ♀20.26–21.00; length of hind tibia: ♂16.30–16.40, ♀17.2–18.4; length of hind basitarsus: ♂3.20–3.92, ♀3.78–4.00; length of ovipositor: 13.44–14.20.

#### Distribution.

China (Yunnan).

#### Remarks.

The female of *E.fossa* Lu, Huang & Bian, 2022 is described for the first time.

## ﻿Discussion

The subfamily Rhaphidophorinae includes eight genera (Cigliano et al. 2024). The genus *Eurhaphidophora* can be distinguished from other genera by the structure of the ninth abdominal tergite and the male genitalia (Gorochov 1999; Lu et al. 2022; Dawwrueng et al. 2023). The other genera differ in the form of the male epiproct or the abdominal tergites (Bian and Shi 2016; Qin et al. 2018). However, the classification of some species remains controversial, such as *Neorhaphidophoralongispinula* (Bian, Zhu & Shi, 2017). Up to now, the classification of the subfamily Rhaphidophorinae is based on morphological characteristics, without molecular evidence. We cannot judge whether the distinguishing characters of the classification system for the genera are appropriate. Moreover, the phylogenetic relationship between genera is still unclear. Further studies on the subfamily Rhaphidophorinae based on more evidence are needed.

## Supplementary Material

XML Treatment for Eurhaphidophora

XML Treatment for Eurhaphidophora dulongjiangensis

XML Treatment for Eurhaphidophora tarasovi doitungensis

XML Treatment for Eurhaphidophora pawangkhananti

XML Treatment for Eurhaphidophora fossa

## ﻿Additional information

### Author ORCIDs

Qidi Zhu https://orcid.org/0000-0001-8187-1286

Fuming Shi https://orcid.org/0000-0002-4885-9012

### Data availability

All of the data that support the findings of this study are available in the main text.
